# Effect of Hot Working Parameters on Microstructure and Texture Evolution of Hot-Deformed Zr-45Ti-5Al-3V Alloy

**DOI:** 10.3390/ma15041382

**Published:** 2022-02-13

**Authors:** Yuanyuan Lei, Ya Yang, Yuanbiao Tan, Wenwei Zhang, Shanshan Wu, Min Ma

**Affiliations:** 1 Guizhou Key Laboratory of Materials Mechanical Behavior and Microstructure, College of Materials and Metallurgy, Guizhou University, Guiyang 550025, China; leiyy512@163.com (Y.L.); yangyaing@126.com (Y.Y.); zww19960916@163.com (W.Z.); swcicily@163.com (S.W.); 2 College of Materials Science and Engineering, Hunan University of Science and Technology, Xiangtan 411201, China

**Keywords:** ZrTiAlV alloy, hot working, recrystallization, microstructure and texture

## Abstract

The effect of hot working parameters on the microstructure and texture evolution of the hot-deformed Zr-45Ti-5Al-3V alloy was studied by the electron backscatter diffraction (EBSD) technique. It was found that a high density of dislocations were generated when the alloy was deformed at 700 °C/0.001 s^−1^ and 800 °C/1 s^−1^. With the increment in hot-deformation temperature and the decrease in strain rate, the dislocation density decreased due to the increase in dynamic recrystallization (DRX) degree. The discontinuous dynamic recrystallization (DDRX) and continuous dynamic recrystallization (CDRX) mechanisms co-existed during the hot working of the Zr-45Ti-5Al-3V alloy at a true strain of 0.7. The texture evolution of the alloy during hot working was characterized and the texture component mainly consisted of {001}<100>, {011}<100>, {110}<112>, and {112}<110> textures. The volume fractions of {001}<100> and {011}<100> textures obviously rose with the reduction in strain rate, whereas those of {110}<112> and {112}<110> textures gradually decreased. At a given strain rate, an increase trend in the volume fraction of the {001}<100> texture was observed with rising hot-deformation temperature, while the volume fraction of the {011}<100> texture first increased and then decreased. An opposite trend was visible in the {112}<110> and {110}<112> texture compared with {011}<100> textures.

## 1. Introduction

The Ti-6Al-4V alloy has been widely used to fabricate the critical parts of compressors, gas turbine aero-engines, and airframes owing to its superior strength and toughness, exceptional workability, and excellent weldability [1,2,3,4]. However, the Ti-6Al-4V alloy exhibits poor tribological properties in extreme environments such as high and low temperature and intense radiation, which results in limited applications of the Ti-6Al-4V alloy in the field of extreme environments [5]. It is well known that Zr and its alloys possess a small atomic number, good corrosion resistance, and long-term stability in the size and performance of the parts in the extreme environment, which is considered a promising structural material serviced in high and low temperature and intense radiation. In recent years, some new ZrTiAlV alloys have been designed based on the Ti-6Al-4V alloy by the addition of Zr element [6,7]. The Zr-45Ti-5Al-3V alloy with high strength is one of new ZrTiAlV alloys [6].

Parts of the Zr-45Ti-5Al-3V alloy are usually fabricated by hot forging or rolling. The mechanical properties of the parts depend on the final microstructure achieved during hot working. It is widely accepted that the microstructure evolution of the metals and alloys is a complex process during hot working [7,8,9]. At the stage of hot working, working hardening is observed. With the increment in strain, dynamic recovery (DRV) and dynamic recrystallization (DRX) may occur. In general, dynamic recrystallization is considered as an effective means to refine the grains of the metals and alloys in industrial production, which is closely connected with hot working parameters. Thus, it is critical to investigate the DRX behavior and microstructure evolution of the new Zr-45Ti-5Al-3V alloy deformed at different hot working parameters.

Recently, numerous research has been executed on the effect of hot working parameters on dynamic recrystallization behavior and microstructure evolution of the metals and alloys [10,11,12,13]. Wu et al. [10] reported that the degree of DRX in the Ti-22Al-25Nb alloy obviously rose with the strain, and the occurrence of dynamic recrystallization can result in a distinct weakening in the deformation textures. Lin et al. [11] found that the flow characteristics of the Ti-55511 alloy was dramatically affected by hot working parameters. Raising the hot-deformation temperature or decreasing the strain rate can accelerate the dynamic recrystallization of β grains. For the Ti-7333 alloy, the main plastic deformation mechanism in the β phase was the dislocation glide owing to their high stacking fault energy [12]. Meanwhile, a significant α-fiber texture including the components of {111} <110> and {112} <110> textures can form after hot working [13].

For the new Zr-45Ti-5Al-3V alloy, in previous work, the hot-deformation behavior of the Zr-45Ti-5Al-3V alloy was investigated [14,15,16,17,18]. The constitutive equation and the processing maps were established, and the optimum processing parameters of the alloy with different initial grain sizes were obtained based on the processing maps. The decrease in initial grain size can greatly reduce the temperature for the full dynamic recrystallization and the optimum processing parameters. Moreover, the final microstructure of the Zr-45Ti-5Al-3V alloy after hot working was obviously affected by hot working parameters including deformation temperature and strain rate. A higher deformation temperature and lower strain rate can give rise to obvious grain-coarsening. In order to control the final microstructure of the Zr-45Ti-5Al-3V alloy after hot working and improve the mechanical properties of the alloy, a deep understanding of the dynamic recrystallization characterization and microstructure evolution of the Zr-45Ti-5Al-3V alloy at various hot working parameters is necessary. Thus, the objective of this paper was to reveal the microstructure and texture evolution of the Zr-45Ti-5Al-3V alloy deformed at various hot working parameters by using EBSD and cellular automata (CA) methods.

## 2. Experimental

**Figure 1:**
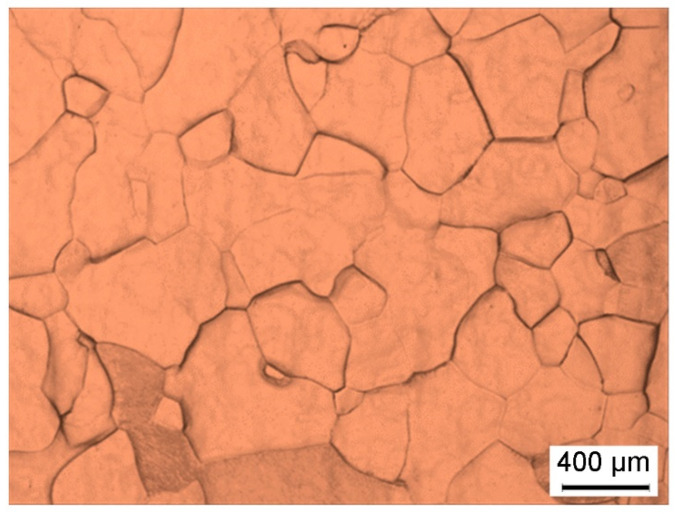
The microstructure of the samples after solution-treatment at 850 °C for 0.5 h.

The Zr-45Ti-5Al-3V alloy used in this paper was a forged rod with a diameter of 45 mm. The T*_β_* temperature of this alloy was measured to be 703 °C [18]. To achieve a full dynamic recrystallization microstructure, the forged rod was annealed at 850 °C for 0.5 h in a tubular vacuum furnace (SG-GS1400, Shanghai Jiejie Electric Furnace Co., Ltd. Shanghai, China), and then water-quenched to room temperature. As shown in Figure 1, a microstructure with an equiaxed recrystallized grain structure was obtained for the heat-treated samples. The average grain size of the heat-treated samples was measured to be 220 μm by Image-Pro software (6, Media Cybernetics, Rockville, MD, USA). Cylindrical samples of Φ8 × 12 mm were machined from the annealed samples. Hot compression tests of the cylindrical samples were operated to characterize the dynamic recrystallization behavior and microstructure evolution of Zr-45Ti-5Al-3V alloys during the hot working. The hot-deformed parameters were designed to be in the temperature range of 700–850 °C with a 50 °C interval and a strain rate range of 1–0.001 s^−1^. All annealed samples were first heated to 850 °C for 10 min, and then cooled to a given hot-deformation temperature at a rate of 20 °C/s for 5 min to reduce the temperature gradient existing in the samples before deformation. The samples were deformed to a given true strain of 0.7, and subsequently water-cooled. The samples for optical microscopy (OM) observation were mechanically polished to a final roughness by metallographic abrasive papers with 300#, 600#, 1000#, 1500#, and 2000#, and chemically etched using a solution of 15% HF + 40% HNO_3_ + 45% H_2_O (Vol.%). The samples for EBSD observation were ground as above and electro-polished in a solution of 70% CH_3_OH + 20% (CH_2_OH)_2_ + 10% HClO_4_ (Vol.%) at the voltage of 25 V for 10 s. The deformed microstructure was characterized by the OM and EBSD technique. EBSD examinations were performed on a Hitachi S-3400N-II scanning electron microscope with an EBSD system. The operating voltage was set as 20 keV and the step size was set at 0.7 μm.

## 3. Results and Discussion

### 3.1. Microstructure Evolution of Hot-Deformed Zr-45Ti-5Al-3V Alloy

**Figure 2:**
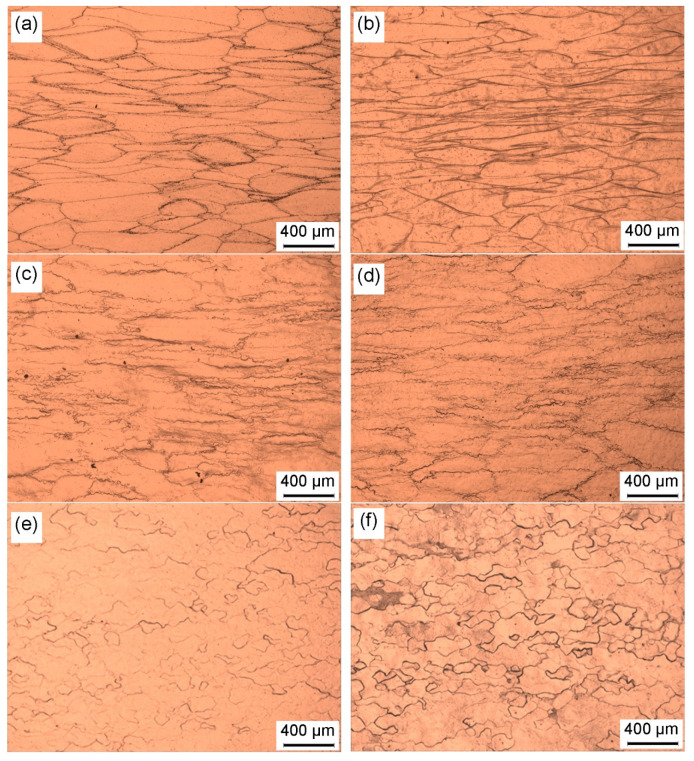
Optical microstructure of the deformed samples under different conditions of (**a**) 700 °C/0.001 s^−1,^ (**b**) 800 °C/1 s^−1^, (**c**) 800 °C/0.01 s^−1^, (**d**) 850 °C/0.01 s^−1^, (**e**) 800 °C/0.001 s^−1^, and (**f**) 850 °C/0.001 s^−1^.

Figure 2 depicts the optical microstructures of deformed samples at different hot working parameters. It is visible from Figure 2a,b that the starting equiaxed grains were stretched perpendicular to the compression direction, and only a bit of fine recrystallized grains formed at the original grain boundaries. The volume fraction of recrystallized grains was calculated to be lower than 5%. At the deformation condition of 800 °C/0.01 s^−1^ (Figure 2c), quantities of fine DRX grains were observed, showing that the dynamic recrystallization took place in the deformed alloy. As the hot-deformation temperature rose and the strain rate decreased, the volume fraction and average size of DRX grains pronouncedly increased (Figure 2d,e). When the hot-deformation temperature was 850 °C, the microstructure with fully recrystallized grains was achieved (Figure 2f).

**Figure 3:**
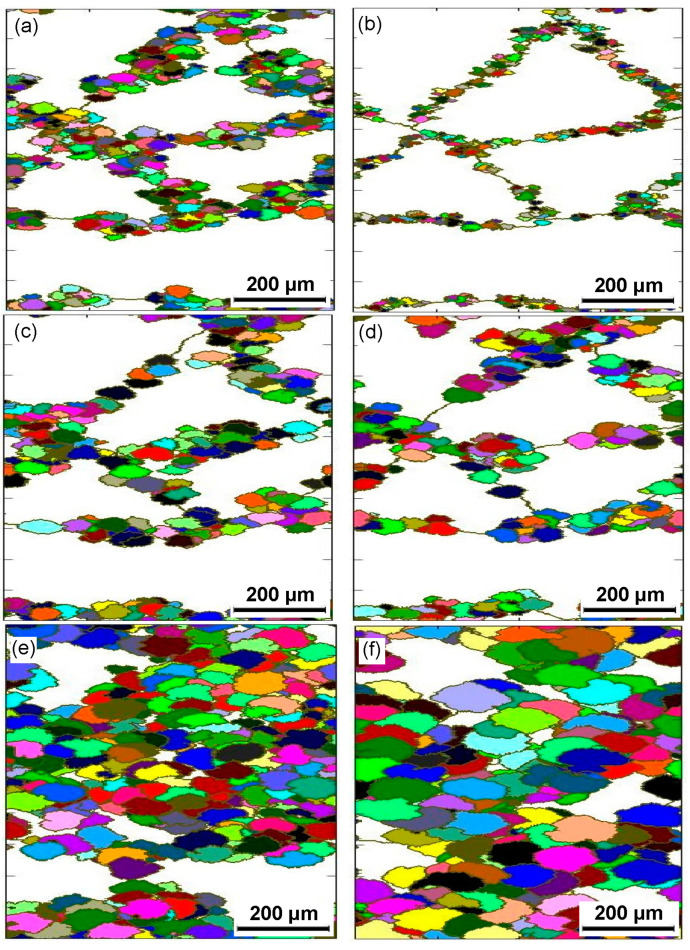
CA maps of the deformed samples under different conditions of (**a**) 700 °C/0.001 s^−1^, (**b**) 800 °C/1 s^−1^, (**c**) 750 °C/0.001 s^−1^, (**d**) 800 °C/0.01 s^−1^, (**e**) 800 °C/0.001 s^−1^, and (**f**) 850 °C/0.001 s^−1^.

In order to further describe the effect of the hot-deformed parameters on the dynamic recrystallization behavior of the Zr-45Ti-5Al-3V alloy, the nucleation and growth process of DRX grains for the alloy deformed under different deformation conditions was also simulated by cellular automata (CA) software according to the dislocation model, as shown in Figure 3. The cell space used in this model was 250 × 200 square cells, which created a size of 8.5 μm, and the simulated area was 2125 μm × 1700 μm. The cell orientation was set to a random number from 1 to 180, and the cell transformation rule was “Moore neighbor.” The dislocation model can be described as [19,20]:

(1) $$\sigma =\alpha \mu b\sqrt{\bar{\rho }}$$

(2) $$\frac{d\rho }{d\varepsilon }=k_{1}\sqrt{\rho }-k_{2}\sqrt{\rho }$$

where α is 0.5; ρ and $\bar{\rho }$ represent the dislocation density and average dislocation density, respectively; *b* represents the Burger’s vector; μ represents the shear modulus; *k*_1_ and *k*_2_ represent work hardening and dynamic softening coefficients, respectively. There is no ρ gradient inside a single grain of the alloy during the hot working. The $\bar{\rho }$ during the CA simulation can be expressed as [21]:

(3) $$\bar{\rho }=\frac{1}{N_{0}}\sum_{i,j}^{i=A,j=B}\rho_{i,j}$$

It was visible that the fine DRX grains mainly nucleated at the original grain boundaries, and then grew toward the interior of original grains (Figure 3a,b). These results showed that the original grain boundaries were the main nucleation site for the dynamic recrystallization. This is because the original grain boundaries have a high grain boundary energy, which favors the nucleation of DRX grains during hot working. Moreover, both sides of the original grain boundaries presented a large strain gradient, which facilitated the growth of DRX grains toward the side of the original grain boundaries with high dislocation density. As the strain rate dropped and the hot-deformation temperature increased, the occurrence of the dynamic recrystallization could be observed inside the deformed grains (Figure 3c–e). It could be observed from Figure 3 that the simulation results showed a good consistency with the experiment results depicted in Figure 2, showing that the DRX process of the Zr-45Ti-5Al-3V alloy could be better-predicted by the CA simulation.

### 3.2. Dynamic Recrystallization Mechanism of Hot-Deformed Samples

**Figure 4:**
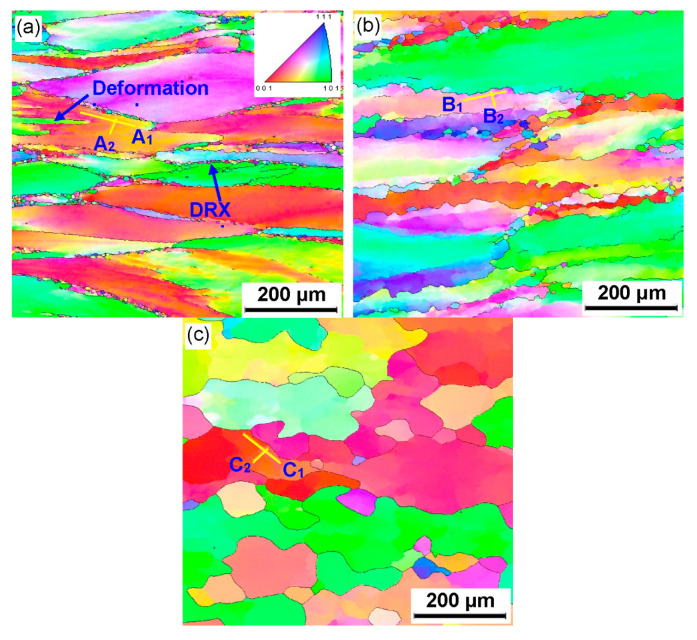
Inverse pole figure (IPF) maps of the deformed samples under different conditions of (**a**) 800 °C/1 s^−1^, (**b**) 800 °C/0.01 s^−1^, and (**c**) 800 °C/0.001 s^−1^.

**Figure 5:**
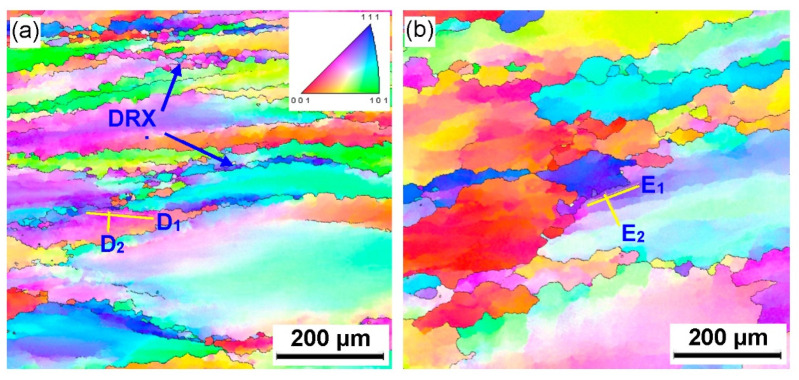

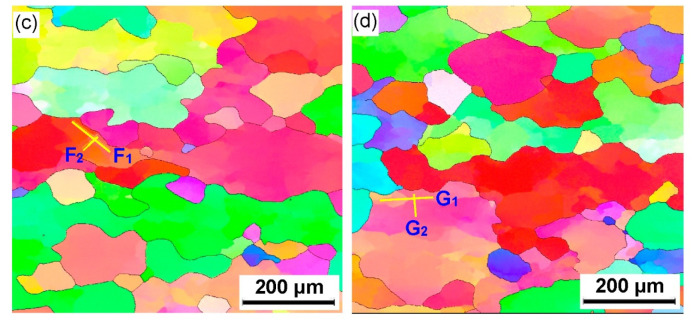
IPF maps of the deformed samples under different conditions of (**a**) 700 °C/0.001 s^−1^, (**b**) 750 °C/0.001 s^−1^, (**c**) 800 °C/0.001 s^−1^, and (**d**) 850 °C/0.001 s^−1^.

**Figure 6:**
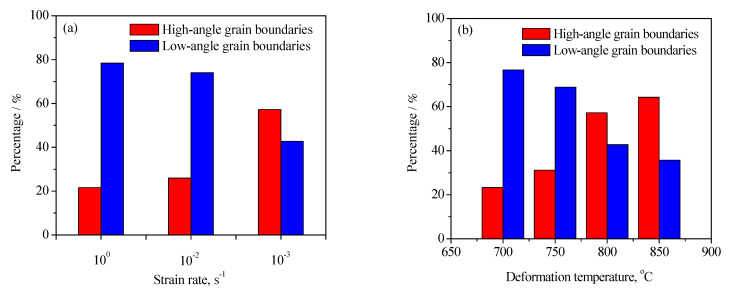
Variation in the volume fraction of high-angle grain boundaries and low-angle grain boundaries with strain rate (**a**) and deformation temperature (**b**).

**Figure 7:**
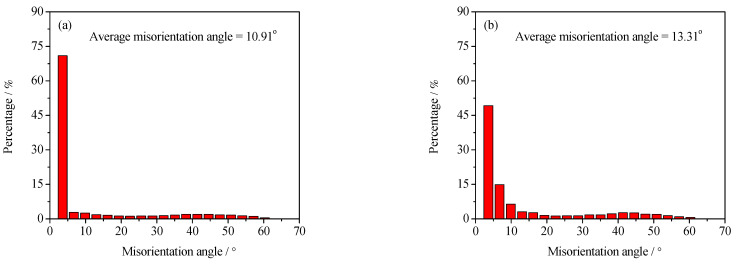

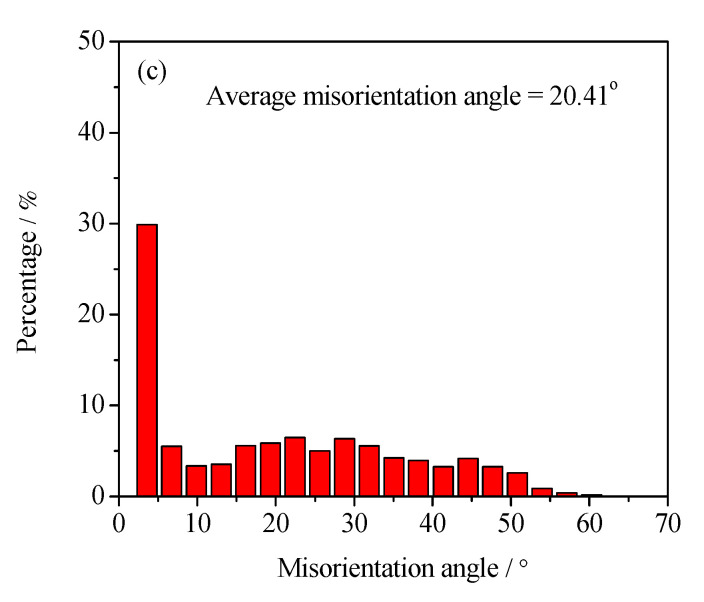
Distribution diagrams of misorientation angles at the deformation temperature of 800 °C and different strain rates: (**a**) 1 s^−1^, (**b**) 0.01 s^−1^, and (**c**) 0.001 s^−1^.

**Figure 8:**
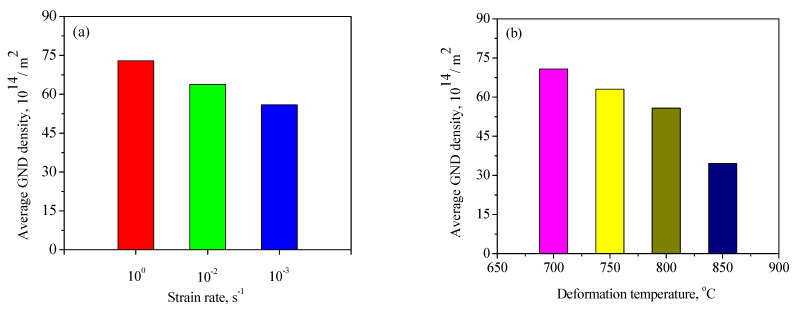
Histogram of dislocation density for the samples deformed at different strain rates (**a**) and deformation temperatures (**b**).

To further analyze the microstructure evolution of the annealed samples in the process of hot working, the microstructure of deformed samples was characterized by using the EBSD technique, as depicted in Figure 4 and Figure 5. The microstructure evolution of hot-deformed samples was dramatically affected by the strain rate and hot-deformation temperature. Figure 4a shows that some deformed grains with serrated grain boundaries were observed and a number of deformation bands were visible in the interior of the deformed grains. The formation of deformation bands resulted from the localized deformation. Usually, the preferred slip system with the lowest deformation energy consumption is easily activated under a higher strain rate condition, which can cause the occurrence of the localized deformation [22,23,24]. Moreover, it can be also seen from Figure 4a that only a bit of fine recrystallized grains nucleated at the grain boundaries of deformed grains, indicating that the dominant deformation mechanism was DRV for the 7Zr-45Ti-5Al-3V alloy deformed at 800 °C/1 s^−1^. It is observed from Figure 6a and Figure 7a that the volume fraction of low-angle grain boundaries (LAGBs, namely 2°–15°) was up to 78.4% and the misorientation angle was mainly in the range of 2°~15° during hot working of the Zr-45Ti-5Al-3V alloy at 800 °C/1 s^−1^. This suggests that a high density of dislocations were produced under the deformation condition of 800 °C/1 s^−1^, which was calculated to be 72.9 × 10^14^/m^2^, as illustrated in Figure 8a. This is ascribed to the fact that the time for the movement of the dislocations and grain boundaries is insufficient under high-strain-rate conditions, which causes a delay in the nucleation and growth of DRX grains. With decreasing strain rate from 1 to 0.001 s^−1^, the volume fraction of LAGBs gradually declined from 78.4% to 42.8% and the dislocation density decreased from 72.9 × 10^14^/m^2^ to 55.9 × 10^14^/m^2^ (Figure 6a and Figure 8a), whereas the average misorientation angle increased from 10.91° to 20.41° (Figure 7). It is noted that the misorientation angle was mainly in the range of 2°~50° and the volume fraction of high-angle grain boundaries (HAGBs) was higher than that of low-angle grain boundaries (LAGBs) at the strain rate of 0.001 s^−1^, as shown in Figure 6a and Figure 7c. This is because that lower strain rate provides enough time, which favors the nucleation and growth of DRX grains. A rise in the volume fraction of DRX grains resulted in a reduction in the dislocation density.

**Figure 9:**
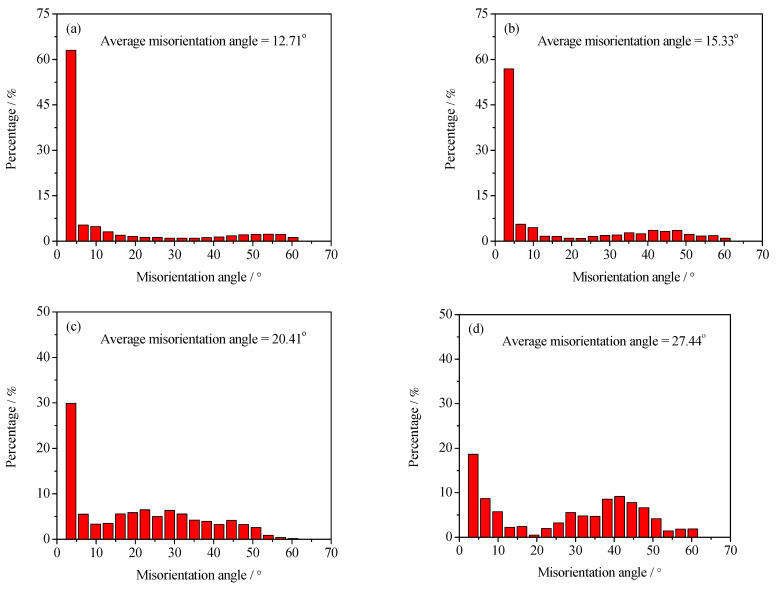
Distribution diagrams of misorientation angles at the strain rate of 0.001 s^−1^ and different hot-deformation temperatures: (**a**) 700 °C, (**b**) 750 °C, (**c**) 800 °C, and (**d**) 850 °C.

It is shown from Figure 5a that the initial grains with serrated grain boundaries were dramatically elongated along the radius direction and a large number of refined DRX grains were visible in the regions near initial grain boundaries and triple points. It can be concluded that the dominant deformation mechanism was DRX for the Zr-45Ti-5Al-3V alloy deformed at 700 °C/0.001 s^−1^. It is illustrated from Figure 6b and Figure 9a that the volume fraction of LAGBs was computed to be 76.7% and the misorientation angle was mainly in the range of 2°~15° and 40°~60°, illustrating that the number of dislocations was generated in the Zr-45Ti-5Al-3V alloy after deformation at 700 °C/0.001 s^−1^, which was calculated to be 70.8 × 10^14^/m^2^ (Figure 8b). With the rise in the hot-deformation temperature, the volume fraction of LAGBs and the dislocation density distinctly declined (Figure 6b and Figure 8b), whereas the average misorientation angle rose from 12.71° to 27.44° (Figure 9). When the hot-deformation temperature rose to 850 °C, the volume fraction of HAGBs was noticeably higher than that of LAGBs and the dislocation density was only 34.6 × 10^14^/m^2^ (Figure 8b). This is due to the volume fraction and size of DRX grains dramatically increasing with the hot-deformation temperature at a given strain rate (Figure 5a–c), which leads to a decrease in the dislocation density. As is well known, the nucleation of DRX grains depends on the generation, interaction, and annihilation of mobile dislocation, while the growth of DRX grains is caused by the migration of the grain boundaries during hot working. At a given true strain, the driving force for the DRX increases with hot-deformation temperature, which accelerates the nucleation and growth of DRX grains [25].

**Figure 10:**
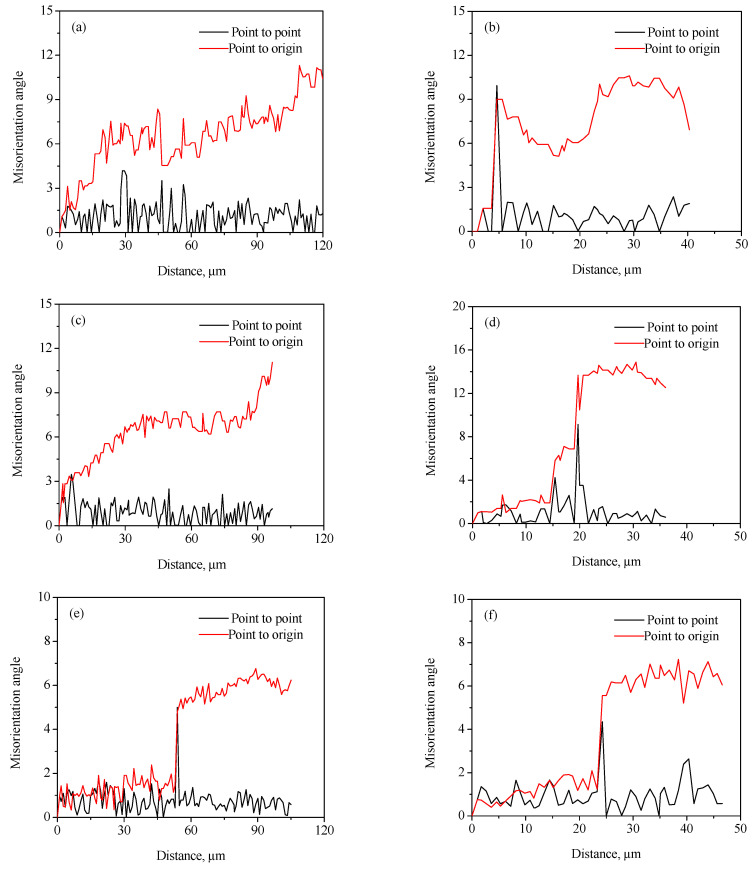
Variation in misorientation angles along the lines marked in Figure 4: (**a**) A_1_, (**b**) A_2_, (**c**) B_1_, (**d**) B_2_, (**e**) C_1_, and (**f**) C_2_.

**Figure 11:**
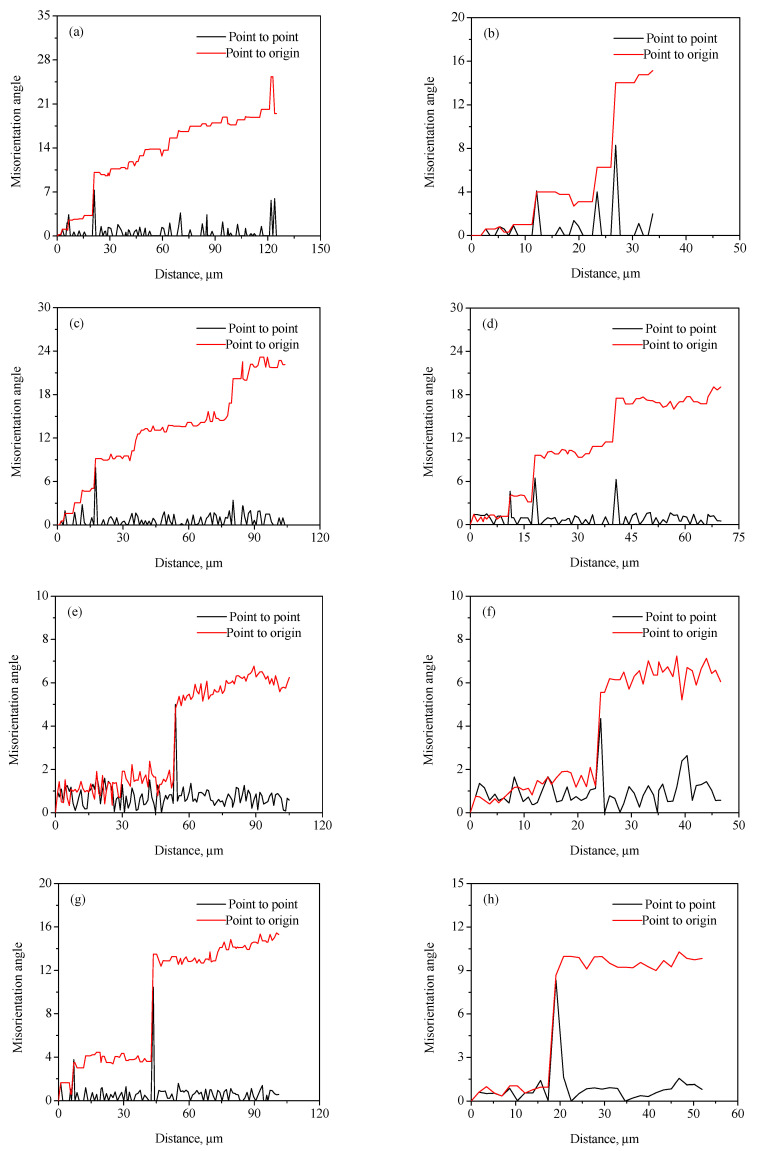
Variation in misorientation angles along the lines marked in Figure 5: (**a**) D_1_, (**b**) D_2_, (**c**) E_1_, (**d**) E_2_, (**e**) F_1_, (**f**) F_2_, (**g**) G_1_, and (**h**) G_2_.

Generally, the discontinuous dynamic recrystallization (DDRX) and the continuous dynamic recrystallization (CDRX) are considered to be the dominating nucleation mechanisms of DRX grains for the hot-deformed alloys [25,26,27,28,29]. For the DDRX mechanism, the recrystallized grains can be produced at the original grain boundaries of deformed grains by the bulging of original boundaries. For the CDRX mechanism, the recrystallized grains can be generated in the interior of deformed grains by the transformation of the formation of subgrains to the progressive rotation of subgrains. Lin et al. [25,27] reported that the change in the cumulative misorientation (Point to origin) and local misorientation (Point to point) was an effective way to characterize the subgrains’ evolution during hot working. In this work, the variations in the cumulative misorientation and local misorientation along the lines marked in Figure 4 and Figure 5 were calculated, as illustrated in Figure 10 and Figure 11. It is observed from Figure 10a,b that the cumulative misorientation along lines A_1_ and A_2_ was higher than 10°. It is suggested that the progressive subgrain rotation occurred in the grain interior, which could promote the development of CDRX [10,22,25,27,28,29]. As the strain rate dropped to 0.01 s^−1^ (Figure 10c,d), the cumulative misorientation along lines B_1_ and B_2_ was higher than 12°, indicating that a large number of the developed sub-grains with LAGBs transformed into the DRX grains with HAGBs. This is ascribed to the fact that more time for the grain boundaries migration can be provided at a lower strain rate, which accelerated the development of the DRX process. As the strain rate reached 0.001 s^−1^, the cumulative misorientation along lines C_1_ and C_2_ was lower than 7°, as depicted in Figure 10e,f. This is associated with the increase in the DRX degree with the increment in strain rate. The deformed grains with high dislocation density were replaced by the DRX grains with free dislocation, which resulted in a decrease in the misorientation angle.

As shown in Figure 11a–d, the cumulative misorientation was higher than 15° both near the initial grain boundaries and inside the grains for the samples deformed at 700 °C/0.001 s^−1^ and 750 °C/0.001 s^−1^. The results showed that the DDRX and CDRX mechanisms co-existed during hot working of the Zr-45Ti-5Al-3V alloy at the lower strain rate and a given true strain of 0.7. When the hot-deformation temperature increased to 800 °C, the cumulative misorientation was lower than 7° both near the original grain boundaries and inside the grains, as illustrated in Figure 11e,f. It was interesting to note that the cumulative misorientation near the original grain boundaries was higher than 15°, whereas the cumulative misorientation within the grains was lower than 10°. This may be attributed to the fact that the DRX grains were deformed again during hot working, resulting in the formation of a large misorientation angle near the grain boundaries of DRX grains.

### 3.3. Texture Evolution of Hot-Deformed Zr-45Ti-5Al-3V Alloy

As is known, grain rotation can occur during the hot working of metals and alloys, which leads to a strong deformation texture before the occurrence of DRX. The deformation texture would gradually be replaced by the new DRX texture after dynamic recrystallization. At a certain strain, the type and volume fraction of texture component mainly relies on strain rate and hot-deformation temperature, which has an obvious influence on the mechanical properties of hot-deformed metals and alloys. Thus, it is necessary to thoroughly understand the texture evolution of hot-deformed metals and alloys. It is generally accepted that the orientation distribution function (ODF) is considered to be an effective way to describe the texture component of the deformed materials [27,28,29,30,31,32,33]. In the ODF sections, each texture component and its intensity can be respectively characterized by the Euler angles g(*φ*_1_, *Φ*, *φ*_2_) and the colored contour.

**Figure 12:**
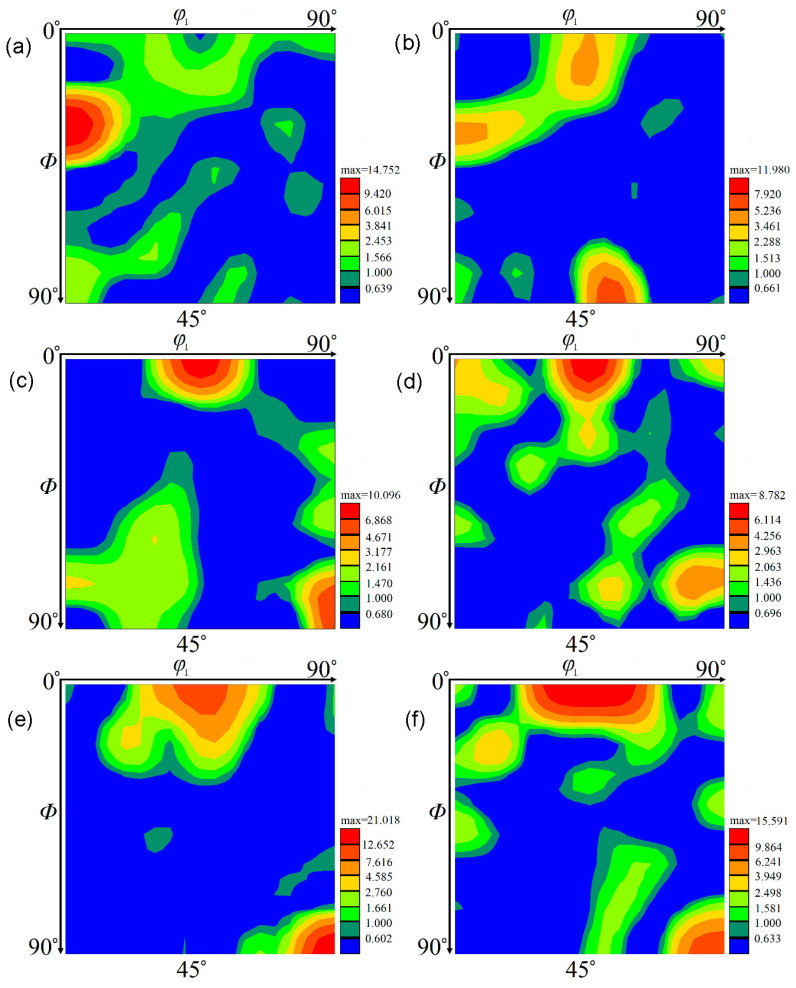
ODF maps of the deformed samples under different conditions of (**a**) 700 °C/0.001 s^−1^, (**b**) 800 °C/1 s^−1^, (**c**) 800 °C/0.01 s^−1^, (**d**) 750 °C/0.001 s^−1^, (**e**) 800 °C/0.001 s^−1^, and (**f**) 850 °C/0.001 s^−1^.

**Figure 13:**
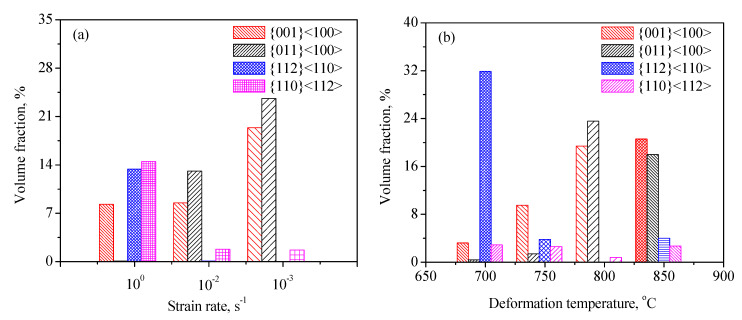
Variation in the volume fraction of different texture components at different strain rates (**a**) and deformation temperatures (**b**).

In this work, the texture evolution of the Zr-45Ti-5Al-3V alloy during hot working was characterized by using the EBSD method. The ODF sections with a constant *φ*_2_ of 45° for the deformed alloy at diverse strain rates and temperatures were drawn based on the measured EBSD data, as exhibited in Figure 12. It is seen that the texture component was mainly made up of {001}<100>, {011}<100>, {110}<112>, and {112}<110> textures. For the body-centered cubic (BCC) alloys, the {001}<100> and {011}<100> textures are considered to be a typical recrystallization texture, whereas the {112}<110> and {110}<112> textures are the dominant deformation textures [10,27,30,31,32,33,34,35,36]. It is shown in Figure 13a,b that the volume fraction of the {001}<100> texture rapidly rose with the reduction in strain rate and increase in hot-deformation temperature. A similar result was also observed in the TB8 titanium alloy [37,38]. This is attributable to the fact that the <100>-oriented grains with a lower Taylor factor can provide a driving force for the grain boundary migration, which accelerates the growth of <100>-oriented grains toward other oriented grains in the DRX process. It is observed from Figure 4 and Figure 5 that the volume fraction and size of DRX grains increased with the decrease in strain rate and the increment in hot-deformation temperature, thus the increasing volume fraction of the {001}<100> texture was observed with the decrease in strain rate and the increment in hot-deformation temperature.

For the {011}<100> texture, it is seen from Figure 13a that the volume fraction of the texture rose with the decrease in strain rate. At a given strain rate of 0.001 s^−1^, the volume fraction of the {011}<100> texture first rose with the increment in hot-deformation temperature and then slightly dropped (Figure 13b). This is associated with the nucleation sites of the {011}<100> texture. Generally, the DRX grains with the {011}<100> texture mainly nucleated in the shear band in the grains, while only a few of the DRX grains with the {011}<100> texture were produced in the grains by the bulging of original grain boundaries [38,39,40]. When the Zr-45Ti-5Al-3V alloy was deformed at a lower temperature, the shear band easily formed, which promoted the nucleation of the DRX grains with the {011}<100> texture. When the Zr-45Ti-5Al-3V alloy was deformed at 850 °C/0.001 s^−1^, the plastic deformation was homogeneous and a full recrystallization with a homogeneous microstructure formed during hot working, which resulted in a reduction in the volume fraction of the {011}<100> texture. For the {112}<110> and {110}<112> textures, it can also be observed from Figure 13a that the volume fraction of both textures dropped with decreasing strain rate. This is related to a rise in the volume fraction of DRX grains with decreasing strain rate, which weakens the intensities of the {112}<110> and {110}<112> texture [10]. At a given strain rate of 0.001 s^−1^, the volume fraction of the {112}<110> and {110}<112> texture first decreased with the increment in hot-deformation temperature and then slightly rose (Figure 13b). This is caused by the DRX grains being deformed again under the deformation condition of 850 °C/0.001 s^−1^, which gave rise to the formation of the {112}<110> and {110}<112> textures.

## 4. Conclusions

In this work, the effect of hot working parameters on the microstructure and texture evolution of the hot-deformed Zr-45Ti-5Al-3V alloy was studied by the EBSD method. The primary conclusions can be drawn as follows:

(1) The strain rate and hot-deformation temperature have an obvious influence on the microstructure and texture evolution of the Zr-45Ti-5Al-3V alloy. At the hot working parameter of 800 °C/1 s^−1^, numerous deformed grains with serrated grain boundaries were observed and a number of deformation bands were visible in the interior of deformed grain. With the increment in hot-deformation temperature and the reduction in strain rate, dynamic recrystallization took place in the deformed alloy and the volume fraction of DRX grains gradually increased.

(2) With the increment in hot-deformation temperature and the reduction in strain rate, the volume of DRX grains gradually increased and a decrease trend in the dislocation density was observed due to the transformation of the developed sub-grains with low-angle grain boundaries transformed into the DRX grains with high-angle grain boundaries. The DDRX and CDRX mechanisms co-existed during the hot working of the Zr-45Ti-5Al-3V alloy at a given strain of 0.7.

(3) For the hot-deformed Zr-45Ti-5Al-3V alloy, the texture component was mainly made up of {001}<100>, {011}<100>, {110}<112>, and {112}<110> textures. At a certain temperature of 800 °C, the volume fraction of {001}<100> and {011}<100> textures obviously rose with the decrease in strain rate, whereas the volume fraction of {110}<112> and {112}<110> decreased. At a certain strain rate of 0.001 s^−1^, the volume fraction of the {001}<100> texture increased with the hot-deformation temperature, while the volume fraction of the {011}<100> texture first increased and then decreased. For the {112}<110> and {110}<112> textures, the volume fraction of both textures first decreased with the increment in hot-deformation temperature, and then slightly increased.

## Data Availability

Data sharing is not applicable to this article.
